# Simulation of Granular Flows and Pile Formation in a Flat-Bottomed Hopper and Bin, and Experimental Verification

**DOI:** 10.3390/ma4081440

**Published:** 2011-08-22

**Authors:** Shinichi Yuu, Toshihiko Umekage

**Affiliations:** 1 Ootake R. and D. Consulting Office, 1-17-27-508 Ootake, Higashiku, Fukuoka 811-0322, Japan; 2 Department of Mechanical Engineering, Kyushu Institute of Technology, 1-1 Sensuicho, Tobataku, Kitakyushu 804-8550, Japan; E-Mail: umekage@mech.kyutech.ac.jp

**Keywords:** granular flow, granular pile, simulation, constitutive equation, flat-bottomed hopper and bin, smoothed particle hydrodynamics, discrete element method, two-way coupling method

## Abstract

Granular flows of 200 μm particles and the pile formation in a flat-bottomed hopper and bin in the presence of air and in a vacuum were predicted based on three-dimensional numerically empirical constitutive relations using Smoothed Particle Hydrodynamics and Computational Fluid Dynamics methods. The constitutive relations for the strain rate independent stress have been obtained as the functions of the Almansi strain including the large deformation by the same method as Yuu *et al.* [1]. The constitutive relations cover the elastic and the plastic regions including the flow state and represent the friction mechanism of granular material. We considered the effect of air on the granular flow and pile by the two-way coupling method. The granular flow patterns, the shapes of piles and the granular flow rates in the evolution are compared with experimental data measured under the same conditions. There was good agreement between these results, which suggests that the constitutive relations and the simulation method would be applicable for predicting granular flows and pile formation with complex geometry including free surface geometry. We describe the mechanisms by which the air decreases the granular flow rate and forms the convergence granular flow below the hopper outlet.

## 1. Introduction

Granular flows through a hopper and granular piles subsequently formed on the bottom of a storage vessel have been topics of extensive research for several decades. These granular flows and piles are used in many kinds of technologies, for example, the handling of minerals, grains, chemicals and pharmaceuticals, feeding solid fuels and mining, and appear widely in the natural environment. The correct elucidation for static and dynamic mechanisms of granular flow and pile formation contributes to the improvement of various production technologies and the prevention of natural geophysical hazards.

Studies of the mechanism of granular flow in which multi-body contacts and multi-body collisions mainly occur are divided into two categories. One is the continuum approach and the other is the discrete particle model. The constitutive relations needed for closure in the continuum approach have been derived by many kinds of models. Recently, many of these studies have been reviewed by Yuu and Umekage [1]. Some other recent studies using the continuum model are as follows: Aranson and Tsimring presented the flows in inclined chutes, avalanches, rotating drums, simple shear cells and some unsteady flows without many fitting parameters using the continuum theory of partially fluidized granular flows [2,3]. They applied dissipative dynamics for the order parameter. Recently, Kamrin and Bazant proposed a stochastic flow rule (SFR) where partial fluidization propagates randomly along slip lines for granular materials [4]. The SFR assumes incipient yield everywhere and is currently used only in quasi-2-dimensional geometries. DorMohammadi and Khoei have developed the three-invariant cap plasticity model based on an isotropic-kinematic hardening rule [5]. The calculated stress ratio and the volumetric strain versus the axial strain in tri-axial test were compared with experimental results and fairly good agreements were observed between them.

For the granular flow in the hopper, Yang *et al.* indicated the influence of the use of elastoplastic non-coaxial model for granular materials on wall pressure prediction in hoppers [6]. The granular material in hopper experiences considerable principal stress rotation. Employing the Nedderman and Tuzun kinematic model [7], Chou *et al.* constructed a boundary-value problem for granular material in a two-dimensional flat-bottomed hopper with eccentric discharge [8]. Drescher and Ferjani presented the evolution of half-width and height of granular layer in flat bottomed bins using the kinematic model [9]. Good agreement between the model and experiment was demonstrated. However the kinematic model would be essentially difficult to apply to the very dense granular material. Vidal *et al.* presented the wall overpressure in the hoppers due to discharge using the Drucker-Prager plasticity model [10]. They showed that, in the case of the flat-bottomed hopper, the overpressure occurred in the lower part of hopper.

On the other hand, a typical discrete particle model is the Discrete Element Method (DEM), which yields simulation results that approximately describe the behavior of granular materials [11]. The multi- body interaction forces among particles are directly calculated in it. Many researchers have studied granular flows in two and three-dimensional hoppers and silos using DEM. They give acceptable qualitative simulation results for real phenomena. For example, Goda and Ebert, Zhu *et al.*, Ahn, Anand *et al.*, Kruggel-Emden *et al.* and Wu *et al.* simulated the granular flows in containers and the granular discharge using DEM [12,13,14,15,16,17]. Ketterhagen *et al.* and Anand *et al.* investigated segregation of granular materials during discharge from hoppers [18,19,20]. Balevicius *et al.* studied the friction effect in filling and discharge of wedge-shaped hopper using DEM [21]. Kruggel-Emden *et al.* simulated the granular flow within the hopper of rectangular design with a prism-shaped bottom using DEM [16]. They compared the two snapshots of the positions of 6mm spheres with the experimental snapshots at t = 2.4 s and 4.2 s after the hopper outlet was opened. Good agreement was demonstrated. Based on DEM simulation, Tuzun *et al.* showed that the great heterogeneity of bulk density exists in the granular pile and stratified layers of relatively dense and loose packing in the pile is evidence of shear bands [22]. Since the possible number of particles for which calculations can be made in DEM is tens of millions or fewer, DEM would not be applicable to simulate directly the mechanism in a real system which consists of a large number of granular particles, particularly small particles with diameters of less than 500 μm.

Thus, the significant prerequisites for a better understanding of the mechanics of granular material are: more appropriate constitutive relations, better simulations to cover the typical phenomena of granular material and experiments for verification of constitutive models and simulation methods. Babic *et al.* derived the phenomenological forms of constitutive equations and presented the transition mechanism between rapid and quasistatic granular flows based on the DEM simulation results of two-dimensional disks [23]. Their results would be used to determine unknown coefficients in the general constitutive equations. Recently, Ji and Shen derived the similar constitutive equations and studied the internal parameters including the contact time, the multiple collision group size and the coordination number for a 3-dimensional simple shear flow of soft poly-dispersed granular materials [24]. The results indicate that a relatively sharp transition between solid-like and gas-like phase exists for low shear rates, when concentration varies from high to low. One of the objectives set by the present authors was to derive constitutive relations based on the mechanical data of granular materials obtained by DEM calculation. The microstructures of individual particles in granular material have been considered in such constitutive relations. It is desirable for the derivation of constitutive relations for a continuum model to use data in mechanical fields which usually appear in granular flows and piles. The data for a huge number of particles are not necessary to calculate constitutive relations. Therefore, uses of DEM data which describe the behavior of various granular particles seem to be suitable for the derivation of constitutive relations for the continuum approach to granular materials. The present authors calculated three-dimensional stresses, strains which are represented by the Almansi strain tensor including the large deformation and bulk density of cohesionless granular material in the same granular stress and strain field of Yuu *et al.* using three-dimensional DEM [1,25]. Based on these data, three-dimensional stress and strain relations, which are the numerically empirical constitutive relations, have been calculated and the numerically empirical constitutive equations for granular materials have been obtained by the same method as Yuu *et al.* [1,25].

The difference between the constitutive relations of the present paper and references [1] and [25] is that the present constitutive relations are the functions of Almansi strain tensor **Ea** and its scalar, on the other hand the constitutive relations of references [1] and [25] are the functions of the infinitesimal strain tensor **γ** and its scalar or the normal and shear infinitesimal strain sizes. **Ea** is rotation invariant. The constitutive relations of the present study are rotation invariant because the constitutive relations of the present study are the functions of Almansi strain tensor **Ea** and its scalar. Thus, the present constitutive relations are objective and applicable to the large deformation. Since the rotation is included in infinitesimal strain tensor **γ**, **γ** is not rotation invariant. Thus the infinitesimal strain tensor **γ** is not applicable to the large deformation in which the rotation frequently occurs. The infinitesimal strain tensor **γ** is not applicable to the present granular flows because there are large deformations in the present granular flows in the hopper and bin.

This paper mainly aims to describe the unsteady, large scale flows of granular materials and the pile formation including the final stage of pile with angle of repose using the present constitutive relations and SPH, and to validate the present constitutive relations. When the slit is opened, the granular material flows out and complex and large-scale flows, including large deformation, occur. Since the unsteady flows have many flow states and many phenomena, the comparison of unsteady flows between calculated and the experimental data gives the detail validation. So we use a large slit which generates unsteady and large-scale flows. The present granular flow from the large slit is a typical unsteady and large scale granular flow and presents detailed information about granular flow. Using SPH and constitutive relations, the detailed unsteady flows and piles of small particles which would be difficult to be simulated by DEM could be simulated.

In this paper, we present simulation results of those granular flows of 200 μm particles and piles in a flat-bottomed hopper and bin for the typical examples of important phenomena for granular material using our numerically empirical constitutive relations and verify our constitutive relations and the simulation method by comparing them with the experimental results. We considered the effect of air on the granular flow and pile by the two-way coupling method which employs Smoothed Particle Hydrodynamics (SPH) method for the granular continuum and computational fluid dynamics (CFD) for the air with an interacting coupling term and present the interaction mechanism between the air and the granular particles in a hopper and a bin.

A brief review and explanation of the SPH method is described in the next chapter.

## 2. Description of Simulation

### 2.1. Granular Flows and Piles in a Flat-Bottomed Hopper and Bin

Yuu *et al.* calculated the granular stress in granular flows using DEM for spherical glass beads and indicated that the stress in the granular material is represented by the strain rate-independent (quasistatic) stress by multi-body contacts and collisions and the strain rate-dependent stress by two body collisions [26]. The calculated granular stresses and the snap shots of granular flow are in fairly good agreement with the experimental results [26]. Yuu *et al.* showed that the strain rate-dependent stress is less than about 5% of the strain rate-independent stress [26], when ( ∂ u_pi_/ ∂ x_j_)(D_p_/g)^0.5^ for i ≠ j is less than about 0.2 and ( ∂ u_pi_/ ∂ x_j_)(D_p_/g)^0.5^ for i = j is less than about 0.04, where ∂ u_pi_/ ∂ x_j_ is the granular velocity gradient, D_p_ is particle diameter and g is gravitational acceleration. Based on the calculated results of the present study, the maximum values of ( ∂ u_pi_/ ∂ x_j_)(D_p_/g)^0.5^ for i ≠ j and ( ∂ u_pi_/ ∂ x_j_)(D_p_/g)^0.5^ for i = j were about 0.2 and 0.05, respectively. Therefore, the above conditions are almost satisfied in the granular flows of the present study. The inertial number I= $\dot{\gamma }$_ij_ D_p_/(p/ρ_p_)^0.5^ for i ≠ j has been considered by Jop *et al.* and da Cruz *et al.* [27,28], where $\dot{\gamma }$_ij_ for i ≠ j is the shear strain rate, p is an isotropic pressure which corresponds to the averaged normal stress τ defined by Equation 8 in the present study and ρ_p_ is real particle density. Da Cruz *et al.* indicated that when I is less than 10^−2^, the granular flow is in the quasistatic regime. The inertial number I of the present study is less than 10^−2^ in about 95% of the present granular flow field except the region below the hopper outlet. Thus the main region of the present granular flow is in the quasistatic regime. Therefore the strain rate-independent stress plays an important role in the present granular flow and the constitutive relations for the strain rate-independent stress are essential for the continuum approach. As shown in Equations 1–6, we assume that the strain rate-independent stress **τ_ps_** in the granular flow is represented by the Almansi strain tensor **Ea** and A1-A6 which are the nonlinear functions of the scalar [Ea_ij_ Ea_ij_]^0.5^ [29], where Ea_ij_ is the component of **Ea**. The summation convention with respect to repeated subscript is used in this study. Equations 1–6 are our numerically empirical constitutive equations and show the components of the strain rate-independent stress, τ_ps, ij_.

τ_ps, xx_ = *f*(ρ_b_)[A1 Ea_xx_ + A2(Ea_yy_ + Ea_zz_) + A3(Ea_xy_ + Ea_xz_)]α (1)

τ_ps, yy_ = *f*(ρ_b_ )[A1 Ea_yy_ + A2(Ea_zz_ + Ea_xx_) + A3(Ea_yx_ + Ea_yz_)]α (2)

τ_ps, zz_ = *f*(ρ_b_ )[A1 Ea_zz_ + A2(Ea_xx_ + Ea_yy_) + A3(Ea_zx_ + Ea_zy_)]α (3)

τ_ps, xy_ = (A4 Ea_xy_ + A5 Ea_xz_ + A6 Ea_zy_)τ (4)

τ_ps, yz_ = (A4 Ea_yz_ + A5 Ea_yx_ + A6 Ea_x__z_)τ (5)

τ_ps, zx_ = (A4 Ea_zx_ + A5 Ea_zy_ + A6 Ea_yx_)τ (6)

τ_ps, yx_ = τ_ps, xy_, τ_ps, zy_ = τ_ps, yz_, τ_ps, xz_ = τ_ps, zx_ (7)

τ = |τ_ps, xx_ + τ_ps, yy_ + τ_ps, zz_|/3 (8)

where ρ_b_ is bulk density of granular material and τ is the averaged normal stress.

Lubliner suggests in his book that the Almansi strain tensor **Ea** is the most commonly used strain tensor for large deformation [29]. The rotation is excluded in **Ea** and A1-A6 are scalar functions of **Ea**. Therefore, the constitutive equations 1–6 are objective [29]. Equation 9 gives **Ea**.

**Ea** = (**I** − **B**^−**1**^)/2 (9)

where **B** defined by Equation 10 is the left Cauchy-Green tensor and **I** is the unit tensor.

**B = FF^T^** (10)

where **F** is the deformation gradient tensor and the superscript T means the transpose. The components of **F** are given by Equation 11.

F_iI_ = ∂x_i_/∂X_I_ (11)

where x_i_ = x*_i_*(X_I_, t), and X_I_ is the initial location at time t = 0.

The components of **B** are given by Equation 12:

B_ij_ = (∂x_i_/∂X_I_)(∂x_j_/X_I_) (12)

Appendix A shows the calculation method of Ea_ij_ using Equations 9 and 12 in SPH.

A scaling factor α in Equations 1–3 is the ratio of the characteristic normal stress τ_a_ for the real field to that τ_aa_ of the criterion field in which the numerically empirical constitutive equations have been calculated, that is, α = τ_a_/τ_aa_. The scaling factor α was taken as the ratio of τ_a_ = (bulk density) (gravitational acceleration) (maximum height of granular layer) = ρ_b_ g h_m_ to τ_aa_ = the maximum vertical normal stress in the criterion field. In the present calculation α = τ_a_/τ_aa_ = (1500) (9.8) (0.0867)/(130) = 9.80 was used.

**Figure 1:**
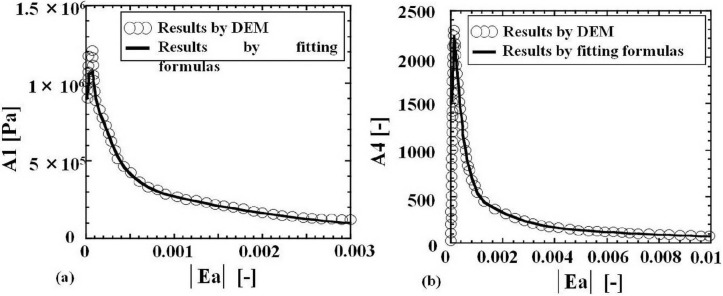
Calculated results of A1 and A4 by Discrete Element Method (DEM) data and fitting formulas in Appendix B. Scalar |**Ea**| is defined as [Ea_ij_ Ea_ij_]^0.5^ = [Ea_xx_^2^ + Ea_yy_^2^ + Ea_zz_^2^ + 2(Ea_xy_^2^ + Ea_yz_^2^ + Ea_zx_^2^)]^0.5^.

**Figure 2:**
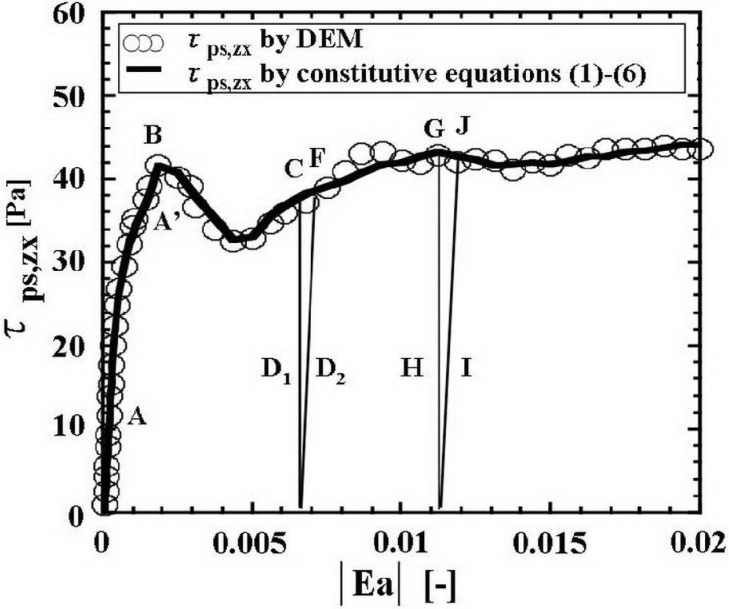
Strain rate-independent shear stress τ_ps,zx_ obtained by the constitutive Equations (1)–(6).

The functions A1–A6 have been obtained by the same method of Yuu *et al.* using the same data of DEM results in the granular stress cell for obtaining constitutive equations of 200 μm particles [1,25]. Yuu *et al.* calculated 16,711 DEM particles which were 200 μm spherical glass beads. The constitutive relations in the present study are specific to the particle size. Strictly speaking, for each particle size a different set of functions A1–A6 should be obtained using DEM. In the present study the constitutive relations for 200μm glass beads were employed to simulate granular flows of 200 μm glass beads. The outline of the method to obtain A1–A6, namely the constitutive equations 1–6, is as follows: As indicated in References [1,25], the components of the stress and strain, and the packing fractions ρ_b_/ρ_p_ of the computational cell in the granular stress field for obtaining constitutive equations were calculated using DEM. When we used DEM data in the stress cell [1,25], the components of the Almansi strain tensor were calculated by the similar method to that shown in Appendix A. In these calculated results, the packing fractions ρ_b_/ρ_p_ of the computational cell in the granular stress field were 0.594–0.604 [25]. The difference of the packing fractions was small. The averaged value 0.6 was used for the calculation to obtain A1–A6 in the present calculation. We used the simple linear equation of state, *f*(ρ_b_) = (5/3)(ρ_b_/ρ_p_). The constant 5/3 was decided by *f*(ρ_b_) = 1.0 at ρ_b_/ρ_p_ = 0.6 in the criterion field of the DEM calculation to obtain the constitutive equations. The scaling factor α is equal to 1, because the stress field is identical to the criterion stress field calculated using DEM for the constitutive equations. Substitution of components of the stress and the strain **Ea** obtained by DEM as mentioned above, the packing fraction, ρ_b_/ρ_p_ = 0.6, and the scaling factor, α = 1, into Equations 1–6 gives the algebraic equations for A1–A6. The solutions give A1–A6 for each stress and strain in the granular material. We assumed that A1–A6 are functions of the scalar |Ea_ij_| only. The results for A1–A6 were plotted in figures of which abscissas are the scalar |**Ea**| = [Ea_ij_ Ea_ij_]^0.5^ of **Ea**. For example, Figure 1 shows the results of A1 and A4. The maximum value was reached in the initial elastic region. Substitution of the functions obtained by fitting A1–A6 into Equations 1–6 gave the numerically empirical constitutive equations. The functions A5 and A6 which were much smaller than A4 were omitted in this paper. All of these fitting functions of A1–A4 are shown in Appendix B. Figure 2 shows τ_ps,zx_ for an example of τ_ps, ij_ obtained using A1–A4 in the stress cell for the constitutive relations. Region **A** in the figure is an initial elastic region and **A^’^** is a transitional region between the elastic and the plastic regions. The plastic deformation starts at point **B**, namely the yielding point. Therefore the reference to a yield condition is not necessary in our constitutive equations. It is obtained automatically. The region **A^’^** and the point **B** show the granular friction process. First the elastic deformation occurs through the process **A**. When the deformation process advances and reaches point **B**, plastic deformation occurs and the granular material flows clearly. If the strain increment becomes negative, in other words the strain decreases, the unloading process takes place, for example, at point **C** and the stress decreases rapidly through process **D_1_**. If the strain increases, the reloading process begins through **D_2_**. Region **D_2_** and point **F** are reloading elastic process and yielding point. Similarly **G**, **H**, **I** and **J** are other examples of unloading and reloading processes. The gradient of **D_2_** is higher than that of **A**. This means that larger stresses, in other words larger forces, are necessary for deformation under the reloading process. Through these processes, the flow velocity decreases and the granular material finally becomes quiescent.

For confirmation, the stresses obtained by the constitutive equations with these functions are compared with the stresses obtained by DEM in Figure 2. It is not surprising that good agreement is obtained. Figure 1 and Figure 2, and Appendix B describe the main features and asymptotic behavior of A1–A4 and the strain rate-independent stress **τ_ps_**.

The strain rate-dependent stress τ_pd, ij_ is the stress due mainly to two body particle collisions and is represented by a function of the strain rate. Almost the same equations as the first terms of Yuu *et al.* [26], which are the basically similar equations to Equation 3 of Jop *et al.* [27], were used for the strain rate-dependent stresses τ_pd, ij_ in this paper. The equations used were as follows:

(13) $${\text{τ}}_{\text{pd},\text{ij}}\;=\;-\text{B}2{\left({\text{D}}_{p}\;/\;\text{g}\right)}^{0.5}\;{\dot{\gamma }}_{\text{ij}}\;\text{τ}\;\text{for}\;\text{for}\;\text{i}\;=\;\text{j}$$

(14) $${\text{τ}}_{\text{pd},\text{ij}}\;=\;-\text{B}1{\left({\text{D}}_{p}\;/\;\text{g}\right)}^{0.5}\;{\dot{\gamma }}_{\text{ij}}\;\text{τ}\;\text{for}\;\text{for}\;\text{i}\;\neq \;\text{j}$$

The constants B1 = 1.67 and B2 = 0.0175 were obtained according to the results of Yuu *et al.* [26].

The SPH method can be extended to simulate many kinds of dense granular phenomena using constitutive relations, particularly granular flows from a container by Sugino and Yuu, granular collapse by Yuu et al, post-failure flows by Bui *et al.* and flows of fractured ice through wedge-shaped channels by Gutfraind and Savage [25,30,31,32]. The details of SPH calculation procedure are omitted here, because Monaghan has described it in detail [33]. The computational domain of continuum granular material is divided into many imaginary (hypothetical) particles which overlap with each other, and the Lagrangian trajectories of these particles with mutual interactions are calculated to describe the mechanical field of the granular material, which is assumed to be a continuum. The distribution of a physical quantity associated with the imaginary particles is given by the kernel function and the value of a physical quantity at a point which is a center of an imaginary particle is obtained by integration over all of the overlapping imaginary particles.

The smoothed value <f(**x**_k_)> for the physical quantity f(**x_k_**) at position **x_k_** in the SPH method is defined as follows:

(15) $$\langle f\left(x_{k}\right)\rangle =\int w\left(x_{k}-x_{l}\right)f\left(x_{l}\right)dx$$

where **x_k_** and **x_l_** are the position vectors of the centers of imaginary particles k and l, and w(**x_k_**− **x_l_**) is the kernel function. ρ_b_(**x_l_**)Δ**x**_l_ = m_0_ shows the relation between the bulk density ρ_b_ and an imaginary particle mass m_0_ , where Δ**x_l_** = Δx_l_Δy_l_Δz_l_ in the three-dimensional case.

The bulk density of granular material is obtained using the continuity equation (mass conservation equation of granular material) transformed as:

(16) $$\frac{d\rho_{b}}{dt}=-\frac{\partial \rho_{b}u_{pi}}{\partial x_{i}}+u_{pi}\frac{\partial \rho_{b}}{\partial x_{i}}$$

where *t* and *u_pi_* are time and granular velocity component.

The interactions of other imaginary particles whose centers are within the sphere of 2h_k_ in radius are taken into account to obtain the smoothed values at the center of particle k [34], where h_k_ = [m_0_/ρ_b_(**x_k_**)]^1/3^ is a distance between imaginary particle centers at **x_k_**. The third order spline functions which are the same formula of Monaghan were used for kernel functions in the present study [33]. Imaginary particles of 20–60 existed in the sphere of 2h_k_ in radius. The rapid approach of imaginary particles towards one another causes an unnecessarily large stress gradient in SPH calculation. Usually, the viscosity term is added to the stresses to prevent the occurrence of an unnecessarily large stress gradient. We used the same formula as Monaghan for this term [33,35].

The governing equation is the three-dimensional momentum conservation Equation 17 of granular material and the continuity Equation 16.

(17) $$\frac{\partial u_{pi}}{\partial t}+u_{pj}\frac{\partial u_{pi}}{\partial x_{j}}=\frac{du_{pi}}{dt}=-\frac{1-\text{Φ}}{\rho_{b}}\left(\frac{\partial P}{\partial x_{i}}+\frac{\partial \tau_{ps,ji}}{\partial x_{j}}+\frac{\partial \tau_{pd,ji}}{\partial x_{j}}\right)+\frac{S_{pi}}{\rho_{b}}-g_{i}$$

Here τ_ps, ji_ is the strain rate-independent stress tensor obtained by the constitutive equations 1–6. The calculation method of **Ea** in the constitutive Equations 1–6 in the granular flow by SPH method is shown in Appendix A. The strain rate dependent stress τ_pd,ji_ is obtained by Equations 13 and 14. In Equation 17, Φ, P, and g_i_ are the void fraction (1 − ρ_b_/ρ_p_), the air pressure, and the component of gravitational acceleration, respectively.

The strain rate independent stress τ_ps, ij_ mainly acts on granular materials which have particles in contact with one another. Yuu and Umekage showed that the strain rate-independent stress acts on granular materials in the region in which the packing fraction ρ_b_/ρ_p_ is larger than about 0.5 in the compression [36]. Therefore, we assumed that, if ρ_b_/ρ_p_ is less than 0.5, the strain rate-independent stress at that point rapidly decreases by the factor [ρ_b_/(0.5ρ_p_)]^96^. This stress is obtained by multiplying the components in Equations 1–3 by [ρ_b_/(0.5ρ_p_)]^96^. For powers of 96 or above, the calculated results were not significantly different. The difference between the simulation results obtained using the 96th power and the 300th power was negligibly small. On the other hand, taking the power below 96 affected the calculated results. This means that taking the power below 96 does not represent the above-mentioned rapid decrease of the strain rate-independent granular stress. Thus, we used the 96th power, namely the factor [ρ_b_/(0.5ρ_p_)]^96^, to represent the rapid decrease of the strain rate-independent stress when ρ_b_/ρ_p_ becomes less than 0.5.

When the increment of normal strain component in the main direction that is vertical in the present calculation becomes negative at a location, we take the location as the end of the loading process and the beginning of an unloading process in the present calculation. The rapid decrease of stress for unloading is calculated using the unloading process constitutive equations which are omitted here. At the end of the unloading process, the point x_i_ was set to the initial point X_I_ for the calculation of the Almansi strain tensor. If the main normal strain increases, the reloading process occurs. The stresses are calculated using the reloading process constitutive equations, which are omitted here. The unloading and the reloading constitutive equations are similar to the initial elastic region of Equations 1–6 for the loading process as shown in Figure 2.

The particle source term S_pi_ on the right-hand side of Equation 17 indicates the mutual interaction between real granular particles and air. When Equations 16 and 17 for the granular flow and the Navier-Stokes Equation 18 and the continuity equation 19 for the air flow are solved simultaneously, the motions of granular material and air are linked through the interaction terms S_pi_ for granular particles and −S_pi_ for air. This is the two-way coupling method [34,37]. Substitution of the drag coefficient into the equation derived by Squires and Eaton [38] gives the equation for S_pi_, which is described in the next section. As shown in Equations 16 and 17, one can solve Equations 16 and 17 as Lagrangian differential equations. The second order Runge-Kutta method is used for the Lagrangian time derivative terms. The trajectories of imaginary particles are obtained. The time step Δt = 2 × 10^−^^6^ s was used for the numerical calculation in order to satisfy the Courant condition based on the speed of sound in the granular continuum using the same method as Gutfraind and Savage [32]. The second-order central difference scheme was used for spatial derivative terms.

### 2.2. Air Flow in a Flat-Bottomed Hopper and Bin

The governing equations for the air flow are the three-dimensional Navier-Stokes equations with interaction terms between the air and particles, and the fluid continuity equation as given in the following equations [39],

(18) $$\frac{\partial \rho \text{Φ}u_{i}}{\partial t}+\frac{\partial \rho \text{Φ}u_{i}u_{j}}{\partial x_{j}}=-\text{Φ}\frac{\partial P}{\partial x_{i}}+\text{Φ}\mu \left[\frac{\partial^{2}u_{i}}{\partial x_{j}\partial x_{j}}+\frac{1}{3}\frac{\partial }{\partial x_{i}}\left(\frac{\partial u_{j}}{\partial x_{j}}\right)\right]-S_{pi}$$

(19) $$\frac{\partial \rho \text{Φ}}{\partial t}+\frac{\partial \rho \text{Φ}u_{i}}{\partial x_{i}}=0$$

Here, u_i_ and P are the air velocity component and air pressure, averaged in the air space. ρ and μ are the air density and air viscosity.

Substitution of the drag coefficient into the equation of the drag force gives the equation for S_pi_. We used Schiller and Naumann^’^s experimental drag coefficient [40], which is applicable to flows of the particle Reynolds number Re_p_ = D_p_|**u** − **u_p_**|ρ/μ < 1000, where |**u** − **u_p_**| is the relative velocity scalar between the air and the real particle. In the present calculation we used D_p_ = 200μm, which is the same as the glass bead diameter for DEM data by which we obtained the constitutive Equations 1–6 and the same as that used in the experiment. The maximum particle Reynolds number in this study was about 150. The equation of S_pi_ is:

(20) $$S_{pi}=3\pi \mu D_{p}\left(1+0.15\text{R}{{\text{e}}_{p}}^{0.687}\right)n\left(u_{i}-u_{pi}\right)\xi \left(\text{Φ}\right)/{\text{C}}_{c}$$

where n is the number density obtained by dividing the bulk density of the granular continuum by the real particle mass, ρ_b_/[(πD_p_^3^)/6]. The correction factor *ξ*(Φ) in the above equation describes the effect of neighboring particles on the drag force. We used the experimental Equation 21 presented by Umekage and Yuu [41]. This equation is applicable to particles of which packing fraction is less than 0.74 for the most densely packing.

*ξ*(Φ) = 3.8 − 5.4/Φ + 2.6/Φ^2^ (21)

**Figure 3:**
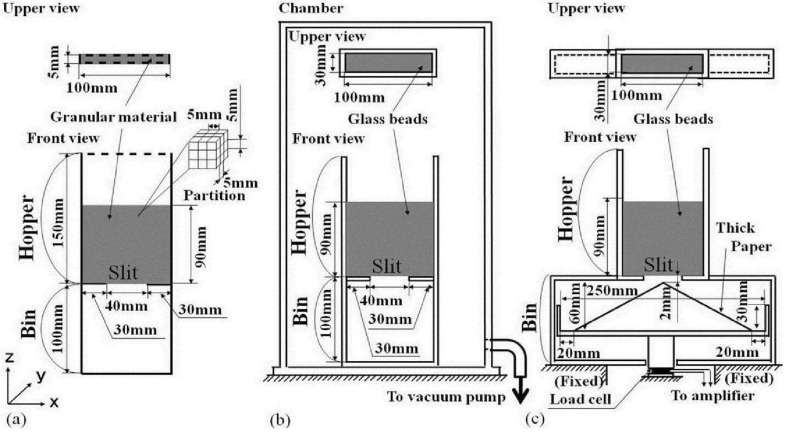
(**a**) Computational domain and boundary conditions; (**b**) Experimental apparatus for snapshot of granular flow; and (**c**) Experimental apparatus for granular flow rate. Bold and dotted lines in (a) show the solid wall boundary conditions (side and bottom walls) and free boundary conditions (top of hopper and front and rear walls of hopper and bin).

The slip factor C_c_ in Equation 20 is 1.0 for a particle of D_p_ = 200μm at atmospheric pressure [42]. The pressure equation derived by taking the divergence of Equation 18 was solved by the relaxation method [43]. Uniform cubic cells were used for the present computation of air flows. The size and the number of air computational cells are 1.25 mm and 64,000, respectively. Since the Reynolds number based on the slit width of the hopper in the present study was less than 2600, the air flows were laminar. In this case, 1.25 mm of the air computational cell size is sufficiently small to represent the air flows in the hopper and bin shown in Figure 3(a) and (b). The fourth-order and the second-order central difference schemes were used for the convection terms and for other spatial derivative terms, respectively, in Equations 18 and 19. The second-order Runge-Kutta method was used for the time derivative terms.

## 3. Computational Domain, Conditions and Procedure

The region above the slit is a hopper and the region under the slit is a bin as shown in Figure 3(a) and (b). The dimensions of the computational domain shown in Figure 3(a) are the same as that of the experimental apparatus shown in Figure 3(b) except the length in the y-direction. Five-millimeter length in the y-direction of the computational domain represents the center region of 30 mm length of the experimental apparatus. We experimentally validated that a length of more than 30 mm in the y-direction of the experimental apparatus did not affect measured granular flow patterns. This means that the effect of the front and the rear glass walls of the experimental apparatus on the granular flow in the center region of 30 mm length in the y-direction of the apparatus is negligibly small. The boundary conditions are shown in Figure 3(a). A partition is 5 mm cubed. We introduce the hypothetical outer computational domain of which width is one partition, 5 mm, along the solid wall and the free boundaries. The imaginary particle configuration in the outer computational domain is symmetric with the imaginary particle configuration in the adjoining part of the interior computational domain. The hypothetical outer particle velocity component perpendicular to the solid wall boundary is set to be opposite to the inner particle velocity component. The other velocity components and the physical quantities of the hypothetical outer particles are equal to those of the inner particles. Twenty seven partitions shown in Figure 3(a) around the reference particle i were used for searching the imaginary particles including the hypothetical outer particles in the interaction range 2 h_i_. When the shear and normal stress ratio, for example |τ_ps,zx_/τ_ps,zz_|, within h_k_ adjoining the bottom and the side walls in the calculation of the granular flow and the pile formation process became less than 0.3, which was the friction coefficient between the granular layer and the solid wall. The velocity parallel to the wall was rapidly decreased to zero by multiplying by 0.95 for each time step Δt. In the SPH method, Lagrangian motions of the imaginary particles are calculated, and thus the free surface is obtained automatically. No constraint is needed at the free surface of SPH calculation. The initial imaginary particle configuration is regular and uniform. The number of imaginary particles and the initial length between the imaginary particle centers are 9000 and 1.67 mm, respectively. The calculation using 21,460 and 1.25 mm has given almost the same results of the granular flows in the same hopper and bin. Thus the imaginary particle number 9000 and the initial length 1.67 mm are reasonable to represent granular flows in the hopper and bin shown in Figure 3(a) and (b). The initial packing fraction is 0.6 which is equal to the averaged initial packing fraction of the experiment. The initial imaginary particle velocity and the initial air velocity in the whole region were zero and the sedimentation of imaginary particles was started under gravity. When the settling velocity of imaginary particles became less than hundreds of micron per second after sufficient sedimentation, we assumed that this was the initial state and the slit at the center of the flat-bottom of the hopper was opened instantaneously. This means that the wall conditions at the slit were removed instantaneously. Then the granular material flowed through the slit and piled on the bottom of the bin. Air flows in the hopper and the bin were generated by the granular flow. The experiment was done similarly. Main computational and experimental conditions are shown in Table 1.

**Table 1:** Computational and experimental conditions.

	Computational conditions.	Experimental conditions.
Particle	Spherical glass beads	Spherical glass beads
Particle diameter, D_p_	200 μm	201 μm
Standard deviation of D_p_	0 μm	6 μm
Particle density, ρ_p_	2500 kg/m^3^	2490 kg/m^3^
Initial particle bed height	83.3 mm	83.3 mm
Initial packing fraction, ρ_b_/ρ_p_	0.6	0.6
Imaginary particle mass of SPH, m_0_	6.94 × 10^−6^ kg	–
Number of imaginary particles of SPH	9000	–
Initial distance between imaginary particle centers of SPH, h_k_	1.67 mm	–
Computational cell sizes of air velocity, Δx = Δy = Δz	1.25 mm	–
Number of computational cells of air velocity, N_x_ × N_y_ × N_z_	80 × 4 × 200 = 64,000	–
Time Step, Δt	2.0 × 10^−6^ s	–

We used a PC, which was a DELL Precision 390 (3.2 GHz), for the computation. It took about 600 hours for SPH and CFD calculation of 1.0 s real time phenomena for the granular flows and piles in the hopper and bin.

## 4. Experimental Apparatus

Figure 3(b) shows the experimental apparatus. Photographs taken by a digital video camera, SONY DCR-TRV20, of which shutter speed is 1/500 s, show the experimental results. A snapshot is obtained per 0.0333 s. The spherical and nearly mono-dispersed glass bead powder was used for the present experiment. The averaged diameter, the standard deviation and the particle density ρ_p_ are 201 μm, 5.6 μm and 2500 kg/m^3^, respectively. These values are almost the same as those used for the present calculation as shown in Table 1. The vacuum pomp (ULVAC KIKO Inc. GVD-200A, Ultimate pressure 0.07 Pa) in Figure 3(b) was used to maintain a vacuum in the apparatus for the experiment in a vacuum. Figure 3(c) shows the experimental apparatus for measuring the granular mass flow rate. As shown in Figure C1 of Appendix C, a plastic plate was inserted to stop the granular flow from the slit. The plastic plate was stopped by a trigger plate and was stretched by four strong rubber bands in Figure C2. The trigger plate was connected to a solenoid valve by a piano wire in Figure C2. The trigger plate was released by the solenoid valve as shown in Figure C3 and the plastic plate was pulled out. After that, the clearance was rapidly choked by the shrinking rubber cement already applied. The action time of the solenoid valve is 0.005 s. A snapshot by the video camera we used is obtained per 0.033 s. The first snapshot showed the stationary plastic plate and in the second snapshot after 0.033 s the plastic plate already disappeared. Therefore the initial state exists at 0.0 s–0.033 s. The accuracy of the video camera is 0.033 s, so we assumed that the second snapshot was the initial state of the flow pattern experiment. The same procedure was done in the experiment of granular flow rate. The granular mass from the hopper outlet was measured by the load cell of which the accuracy is 10^−3^ N, Kyowa Electronic Instrument, during 1.0 s using 10 ms sampling time. The time derivative of the granular mass gives the flow rate. As shown in Figure 3(c), the thick paper of which top is near the hopper outlet was set to reduce the momentum of granular flow.

## 5. Results and Discussion

### 5.1. Granular Flows and Piles in a Flat-Bottomed Hopper and Bin

**Figure 4:**
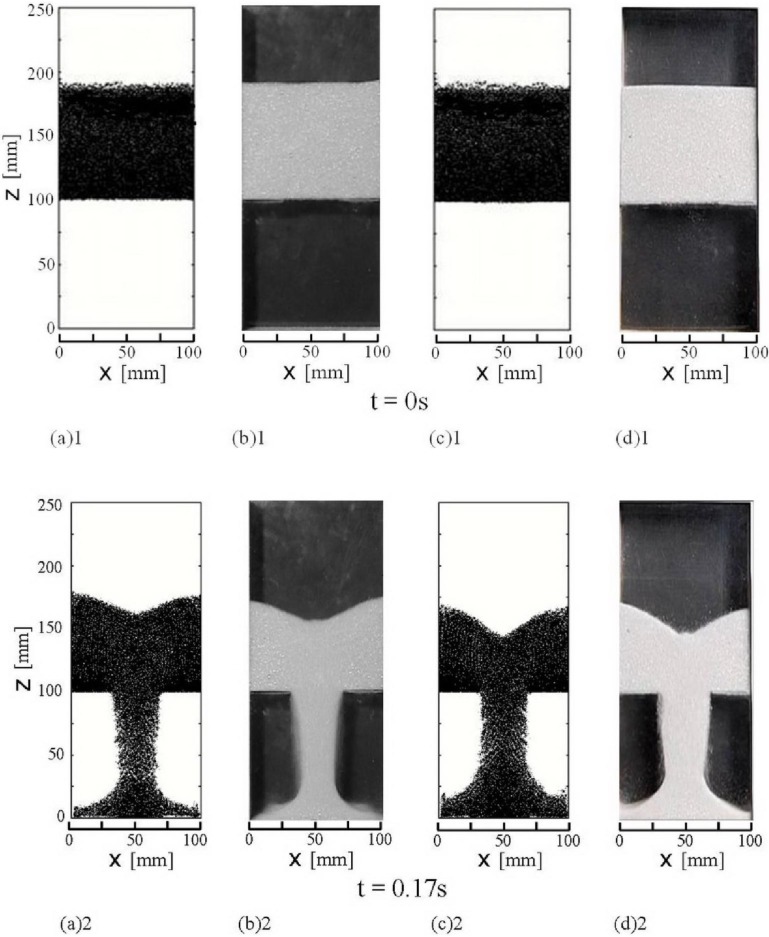

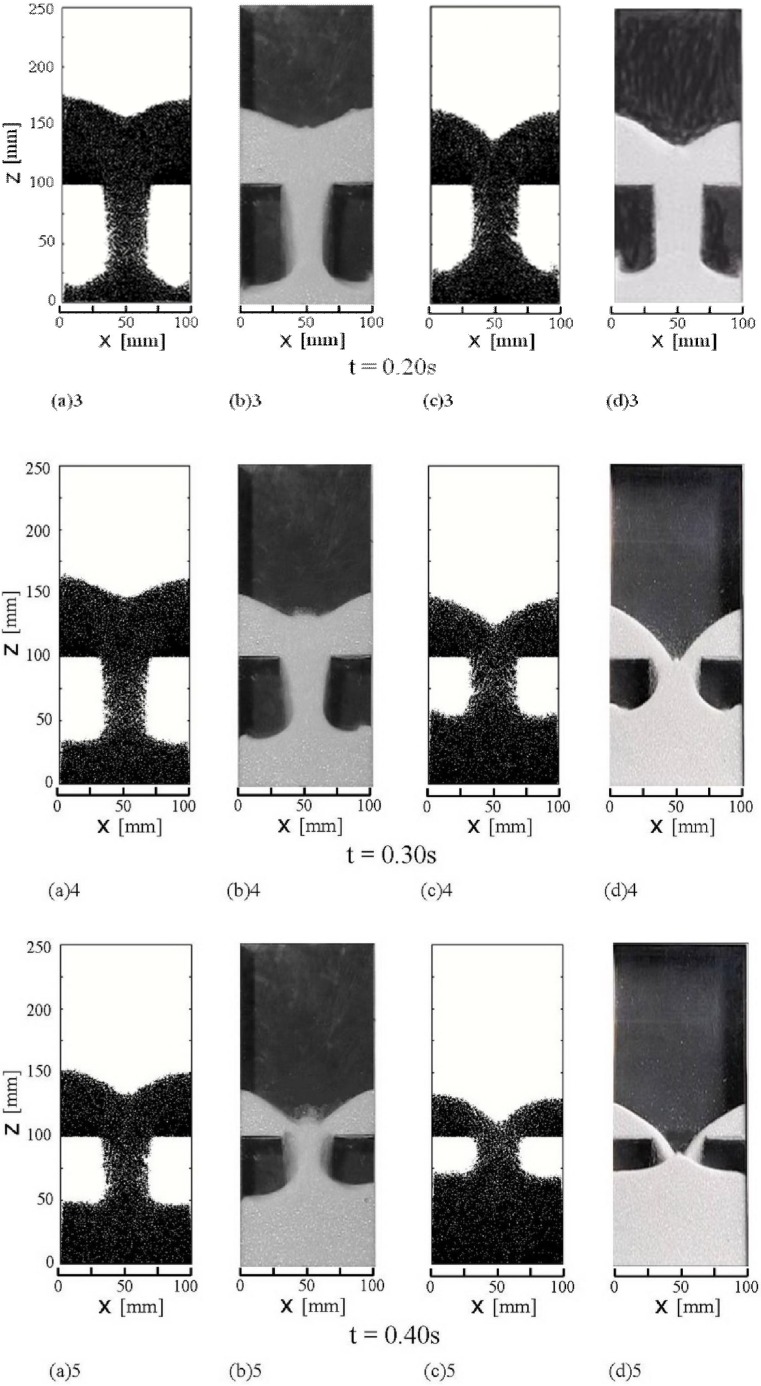

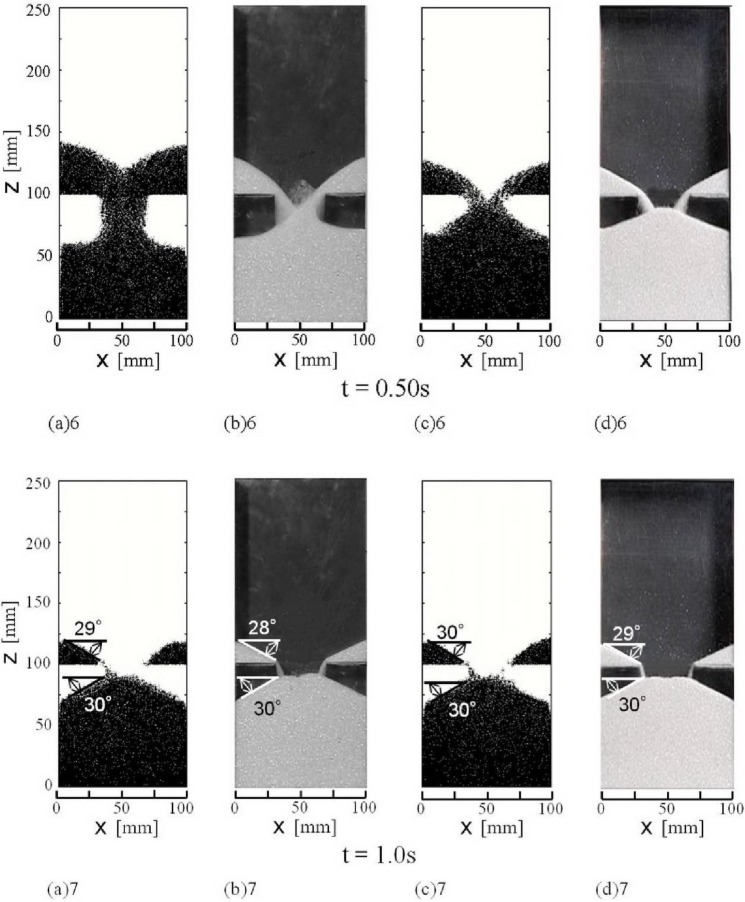
Calculated and experimental snapshots of granular flow in the flat-bottomed hopper and bin. (a)1, (a)2, (a)3, (a)4, (a)5, (a)6 and (a)7 Calculated results in the presence of air. (a’)2 Granular flow areas from the slit outlet to the top of the pile on the bottom of (a)2. (b)1, (b)2, (b)3, (b)4, (b)5, (b)6 and (b)7 Experimental results in the presence of air. (c)1, (c)2, (c)3, (c)4, (c)5, (c)6 and (c)7 Calculated results in a vacuum. (c’)2 Granular flow areas from the slit outlet to the top of the pile on the bottom of (c)2. (d)1, (d)2, (d)3, (d)4, (d)5, (d)6 and (d)7 Experimental results in the very low pressure (10Pa) air. The movie of these granular flows is available at the supplementary material of this paper.

Figure 4 shows the comparison of the calculated granular flow patterns and piles with the experimental results in the flat-bottomed hopper and bin. Dots in the calculated results in the figures indicate 50 dots per one imaginary particle distributed randomly in a sphere of radius 2h_k_, interacting range, in the continuum of granular materials using normal random numbers with the standard deviation = (2/3)h_k_ for visualization of the calculated results, where h_k_ is a distance between imaginary particle centers. We plotted dots around centers of all imaginary particles existing in the computational domain. In this paper, the presence of air means the presence of air under the atmospheric pressure and the very low pressure means 10 Pa of which state is nearly a vacuum. Figure 4 (a)1 and (b)1 show the calculated and the experimental initial states in the presence of air, and Figure 4 (c)1 and (d)1 show the initial states in a vacuum, respectively. Figure 4 (a)2–(a)4 and Figure 4 (b)2–(b)4 show the calculated and the experimental granular flow patterns in the presence of air, and Figure 4 (c)2–(c)4 and Figure 4 (d)2–(d)4 show the calculated and the experimental granular flow patterns in a vacuum at t = 0.17, 0.2 and 0.3 s after the slit is opened, respectively. The slope of the top free surface of the granular flow in the hopper in a vacuum is steeper than that in the presence of air. The stripes that appeared alternately in the calculated granular flow patterns through the hopper outlet indicate the high and low bulk density bands. The granular flow areas from the slit outlet to the top of the granular pile on the bottom of bin in Figure 4 (a)2 and (c)2 were magnified in order for the stripes to be recognizable and shown in Figure 4 (a’)2 and (c’)2. These stripes are not clear in the experimental snapshots of granular flow patterns. The high and low bulk density bands would be caused by the dynamic arches like the stick slip flow which formed in the upper stream on the hopper outlet. The calculated and the experimental flow patterns shown in these figures indicate that the granular flow reaches the bottom of the bin and the granular material begins to pile on the bottom of the bin. The stripes which indicate the periodic fluctuation in the calculated granular flow continues downstream to the top of the pile. The width of the granular flow in the presence of air through the outlet becomes narrower as it flows downstream as shown in Figure 4 (a)2–(a)4 and Figure 4 (b)2–(b)4, namely, the granular flow below the hopper outlet is converged. This is the convergence flow from the hopper outlet. It is not clear what causes the convergence flow. Figure 4 (c)2–(c)4 and Figure 4 (d)2–(d)4 show that the convergence flow does not arise in the granular flows in a vacuum and in the very low pressure. Therefore, the air would primarily cause the convergence flow. We discuss the mechanism later. Flow patterns in the hopper show the funnel flow which is such that the granular material above the slit is mainly in motion and there are large quasistatic regions on both sides of the hopper bottom. The granular flow through the outlet has collided with the bottom of the bin and separated to both side walls of the bin. The granular material that has impacted on side walls slightly climbs along them. The size of pile on the bottom of the bin in these figures shows that the granular flow rate in the presence of air is smaller than that in a vacuum. As discussed later, the interaction between granular particles and the air flow reduces the granular flow rate. A small swelling appears on the center of the top surface of the granular flow in the hopper. Both figures of the granular flows in the presence of air and in a vacuum show the small swelling. The observation of the granular flows suggests that the collision of the granular flows from both sides of the top surface in the hopper would raise the granular particles and form the small swelling at the center of the top surface.

Figure 4 (a)5, (b)5, (c)5 and (d)5 show that more than half of the granular material has flowed out through the hopper outlet at t = 0.4 s. The free surface on the pile in the bin is nearly horizontal and does not form the angle of repose. The pile grows with the similar shape to those at t = 0.2 s and 0.3 s. The shape of pile at t = 0.5 s in the presence of air is still similar to those at t = 0.2s–0.4s as shown in Figure 4 (a)6 and (b)6, but on the other hand the pile in a vacuum is forming the angle of repose because the granular flows from the hopper outlet become quite small and the streams on the free surfaces on the pile in the bin are small as shown in Figure 4 (c)6 and (d)6. Figure 4 (a)7, (b)7, (c)7 and (d)7 show that the flow patterns and piles of granular material at t = 1.0 s are nearly the final flows and shapes of piles in the hopper and the bin. The calculated velocities of granular flows except on the free surfaces and in the small flows from the hopper outlet are less than about 400 μm/s. Therefore, the granular material is almost quiescent. The angles of repose obtained from the calculated piles as shown in Figure 4 (a)7 and (c)7 are about 30 degrees (=0.52 rad) which are equal to the experimental angles of repose measured from the experimental piles in Figure 4 (b)7 and (d)7. Figure 4 shows that the calculated time evolution of granular flow patterns, the sizes and shapes of piles in the hopper and in the bin in the presence of air and in a vacuum are in fairly good agreement with those of the respective experiments. The presence of air largely affected the granular flow of 200 μm particles and changed the whole granular flow pattern.

**Figure 5:**
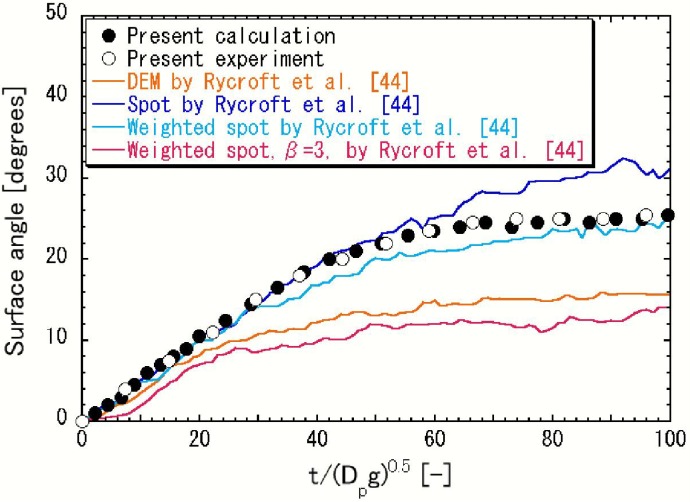
Time evolution of the free surface angle.

The simulation and experimental results of the granular flows and piles of small particles and large outlet are rare at the present stage. Thus, the direct comparison of the present results with other results which are dependent upon the geometry of hopper and bin, particle size and other factors is difficult. The time evolution of surface angles, of which dependency upon the geometry of hopper and bin, particle size and others would be comparatively small, of the present simulation and experimental results is compared with the results of DEM, Spot, Weighted spot and Weighted spot, β = 3 by Rycroft *et al.* [44] in Figure 5. In this figure, the initial state (t = 0) is the state when the flat free surface begins to incline. The results show that the free surface angles increase with increasing time. The simulation result of Weighted spot, β = 3 correspond well with the results of DEM by Rycroft *et al.* It is unclear whether the parameter of biasing, β = 3, is widely applicable to various granular flows with free surface. The present simulation results of the evolution of surface angle averaged on the free surface is in good agreement with our own experimental results. The initially flat free surface becomes more inclined and the angle gradually increases to the angle of repose as shown in Figure 4.

In the present model, the diffusion of particles is not considered. However, Rycroft *et al.* [45] showed that the particle diffusion length is a linear function of particle diameter. Since the particle diameter (=0.2 mm) of the present study is small, the diffusion described in the spot model does not seem to be very large. The diffusion-like phenomena generated by the nearly periodical fluctuation of particle velocity by the stick-slip would not be so small.

**Figure 6:**
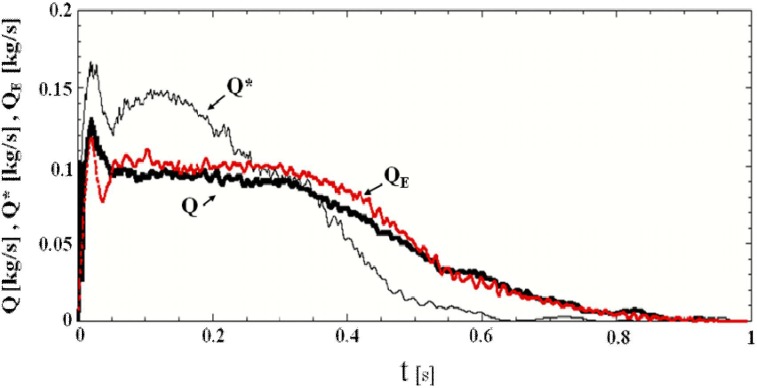
Evolution of granular flow rates from the hopper outlet, where Q, and Q^*^ are the calculated granular flow rates in the presence of air and in a vacuum, respectively, and Q_E_ is the experimental granular flow rate in the presence of air.

**Figure 7:**
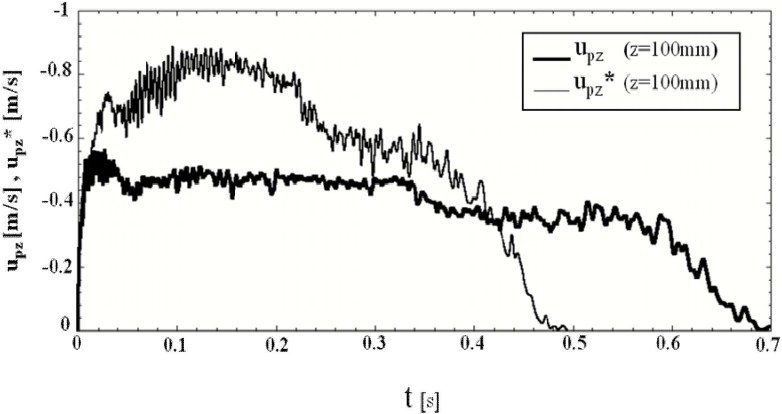
Evolution of calculated vertical granular velocities at the center of slit, u_pz_, in the presence of air and, u^*^_pz_ in a vacuum. The granular velocity is a calculated value averaged in an air computational cell.

**Figure 8:**
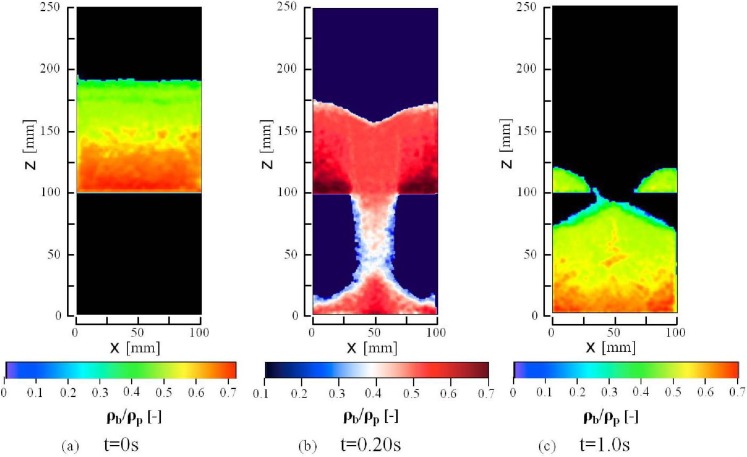
Calculated iso-contour of packing fraction *ρ*_b_/*ρ*_p_ in the presence of air in the center cross-section of the computational domain y = 2.5 mm. The packing fraction is the calculated value averaged in the air computational cell.

**Figure 9:**
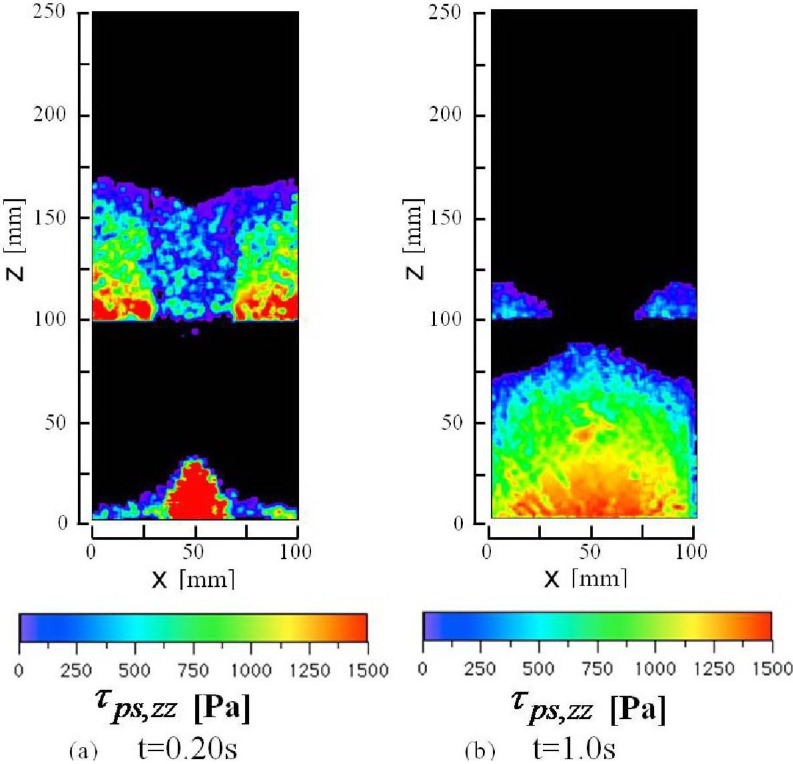
Calculated iso-contour of vertical normal stress (strain rate independent stress), τ_ps,zz_ in the presence of air in the center cross-section of the computational domain y = 2.5 mm. The stress τ_ps,zz_ is a calculated value averaged in an air computational cell.

Figure 6 shows the evolution of the calculated granular flow rates from the hopper outlet in the presence of air Q and in a vacuum Q^*^ and the experimental granular flow rate in the presence of air Q_E_. At the present stage, measuring the granular flow rate in a vacuum has been difficult for us. Thus, we have not measured the granular flow rate in a vacuum. The granular flow rate in the presence of air is nearly constant during t = 0.05 − 0.33 s except for the period shortly after opening the slit. It decreases gradually because a sufficient amount of granular material to maintain the nearly constant flow rate does not exist in the hopper. The evolution of the flow rate Q is in fairly good agreement with the experimental results Q_E_. Figure 6 indicates that the granular flow rate in a vacuum Q^*^ is larger than that in the presence of air Q during the period t < 0.3 s in which a sufficient amount of granular material in a vacuum exists in the hopper. The maximum granular flow rate in a vacuum is about 1.5 times larger than that in the presence of air at t = 0.12 s. The mechanism of the difference by the interstitial air is discussed later. The granular flow rates Q, Q_E_ and Q^*^ reached extreme values shortly after opening the slit, around t = 0.02 s, as shown in Figure 6. The evolution of the calculated vertical granular velocity at the center of the hopper outlet in Figure 7 shows the similar extreme value around t = 0.03 s. In this paper, the granular velocity, the bulk density and the stress calculated using SPH shown in Figure 7, Figure 8, Figure 9 and Figure 12 are averaged values in an air computational cell. The high bulk density region was formed at the bottom of the hopper by the sedimentation of granular particles before opening the slit as shown in Figure 8(a). When the slit is opened, the vertical granular velocity u_pz_ is accelerated by gravity. The maximum of the product, ρ_b_u_pz_, which is the granular flow rate per unit area, was reached shortly after opening the slit, namely at about 0.02 s. After that, the bulk density at the slit decreased because the very high bulk density region in the hopper bottom flowed out and the granular flow rate decreased. Hence, the maximum of u_pz_ was reached at 0.03 s, which is slightly different from 0.02 s, as seen above. After 0.03 s, the growing granular stress by the deformation decreases u_pz._ After that, another deformation process would decrease the granular stress and increase u_pz._ as shown in Figure 7. The experimental vertical granular velocity above the hopper outlet center in the plane hopper measured by Sielamowicz *et al.* showed nearly the same extreme value shortly after opening the slit [46]. Figure 6 shows that the granular flow rates Q, Q_E_ and Q^*^ fluctuated. The detailed calculated results in the presence of air gave that the frequencies of the granular flow rate fluctuation at the hopper outlet at z = 100 mm, the bulk density fluctuation, the vertical granular velocity fluctuation, the vertical normal stress τ_ps,zz_ fluctuation and the scalar of the Almansi strain tensor **Ea** fluctuation at x = 50 mm and z = 105 mm slightly upper center of hopper outlet are 0.0058 s, 0.0066 s, 0.0065 s, 0.0061 s and 0.0071 s, respectively. These are almost the same frequencies. The similar results were obtained in a vacuum. This suggests that the stick-slip like fluctuations of stress and strain in the granular flow in the hopper would cause these fluctuations. Figure 7 shows that the amplitude of granular velocity fluctuation in a vacuum is larger than that in the presence of air. This would be because the air drag force stabilizes the motion of particles in the granular flow.

The fairly good agreement shown in Figure 4 and Figure 6 suggests that our constitutive relations of Equations 1–6 represent the mechanics of granular materials in the hopper and bin.

Figure 8 shows calculated iso-contours of the packing fraction, ρ_b_/ρ_p_, in the presence of air at 0 s (the initial state), 0.2 s and 1.0 s after the slit is opened. Figure 8(b) shows high and low bulk density bands in the granular flow below the hopper outlet as shown in the calculated granular flow patterns in Figure 4. High bulk density regions are formed near both side corners in the bottom of the hopper at t = 0.2 s and the bin at t = 1.0 s. The bulk density in the center region of the pile on the bottom of the bin is slightly lower than those in the other regions on the bottom at t = 1.0 s. The granular flow toward the side walls of the bin has reduced the bulk density in the center region of the pile and the side walls which dammed up the granular flow has increased the bulk density in the side corners in the bin. As shown in Figure 8, the bulk density changes remarkably between the hopper and bin. Figure 9 shows the calculated iso-contours of the vertical normal stress component of the stress independent of the strain rate in the presence of air at 0.2 s and 1.0 s after the slit is opened. Since the packing fraction in the region below the hopper outlet is less than 0.5, the stress, independent of the strain rate, does not act on the granular flow in this region. The results show that there are many stripes and spots of the stress iso-contour. The elastic and the plastic deformations in the deformation process, including the stick slip and the unloading and reloading processes, would mainly cause the sharp local changes and form the complex iso-contours. The calculated horizontal normal stress showed the similar iso-contours, omitted in this paper, to those of the vertical normal stress. The values of the horizontal normal stress were roughly two thirds of the vertical normal stresses.

The weight of the granular material in the hopper and the bin at the nearly final stage, t = 1.0 s in Figure 4 (a), is equal to the total sum of the calculated forces acting on the bottoms of the bin and the hopper, the side walls and the front and the rear surfaces within the difference of about 2%.

### 5.2. Effect of Air on Granular Flow in a Flat-Bottomed Hopper and Bin

**Figure 10:**
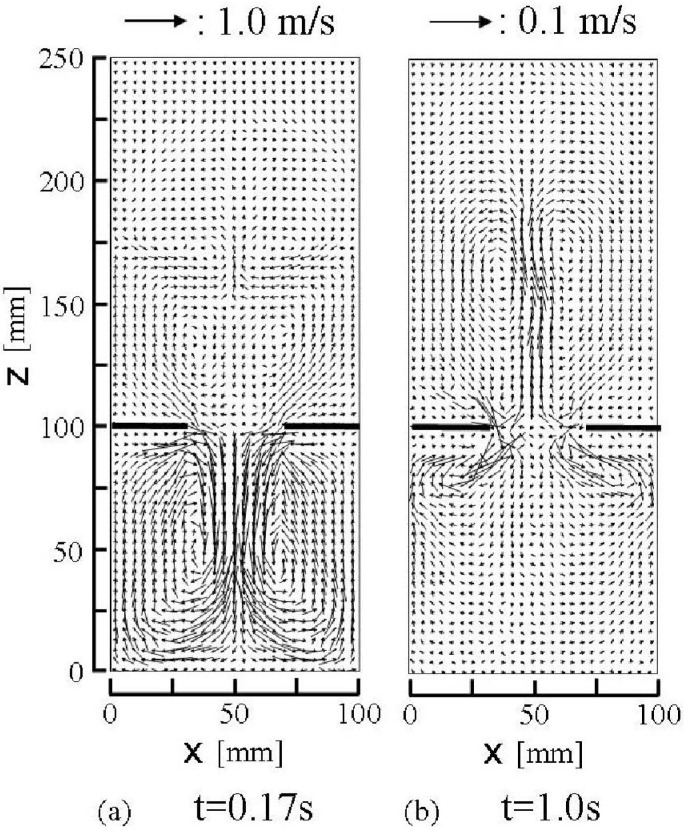
Calculated air velocity vector diagrams in the presence of air in the center cross-section of the computational domain y = 2.5 mm. The point of the arrow head and the length of the arrow indicate the position in the flow field and the size of the velocity vector, respectively.

**Figure 11:**
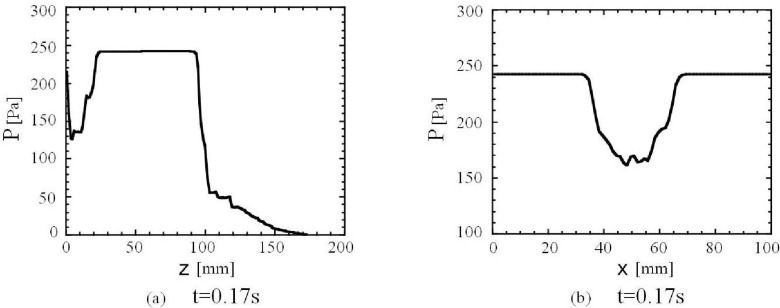
Distributions of air pressure P. (**a**) Vertical distribution at t = 0.2 s and x = 31 mm that is near the edge of hopper outlet and y = 2.5 mm. (**b**) Horizontal distribution at t = 0.2 s and 50 mm height from the bottom of the bin and y = 2.5 mm.

The effect of interstitial air in an open silo was investigated experimentally by Pennec *et al.* and that in an hourglass was done experimentally by Veje and Dimon and Mute *et al.* [47,48,49]. Pennec *et al.* experimentally showed that the dilation of granular material and an interaction with interstitial air cause the silo hiccups. Figure 10 shows calculated air velocity vector diagrams in the x–z plane at t = 0.17 s and 1.0 s. Air flows in the hopper and the bin are generated by the descending granular flow from the hopper outlet. The air is dragged by the descending granular particles, and the air flows impact the bottom of the bin, ascend along the walls of the bin and form the large vortices. When a sufficient amount of granular material exists in the hopper, the air is sucked into the granular flows (shear zone) in the hopper from the edge of the hopper outlet as shown in Figure 10(a). The calculated vertical distribution of air pressure at the horizontal location x = 31 mm that is near the edge of the hopper outlet at t = 0.17 s is shown in Figure 11(a). The result clearly indicates the rapid pressure decrease near the hopper outlet (z = about 100 mm). At the initial state, t = 0, only the air exists in the bin. When granular material flows out from the hopper outlet to the bin, the air flows up from the bin to the hopper owing to the mass conservation of air. The air is sucked from the edge of the hopper outlet as shown in Figure 10(a). The sucked air creates the inflow which ascends through the granular material in both sides of the hopper and forms large pair vortices in the granular material in the hopper as shown in Figure 10(a). These large pair vortices create another large pair of air vortices on the free surface of granular material in the hopper. The region in which the descending velocities of air are small in the central region of the hopper as shown in Figure 10(a) is the plug flow zone. As the granular material flows out from the hopper, the plug flow zone on the hopper outlet decreases, elapsing after the initial state and finally disappearing, as shown in Figure 10(b) and Figure 4(a)7–(d)7. The air flow pattern drastically changes after the disappearance of the plug flow zone. As shown in Figure 10(b), the air descends along the piles in the hopper and ascends from the center region of the hopper outlet. These air flows form a large pair of vortices in the hopper. The air flow in the bin also forms large pair vortices on the pile in the bin.

As mentioned earlier, the calculated granular flow patterns in the presence of air well describe the convergence flow in the measured flow patterns at the presence of air as shown in Figure 4. There is not a clear convergence flow through the hopper outlet in the calculated and the experimental granular flows in a vacuum and in the very dilute air as shown in Figure 4. Figure 11(b) shows that the air pressure outside the granular flow is higher than that inside the granular flow in the bin. The air pressure decreases toward the center of granular flow. The interaction between granular particles and air creates both flows of particles and air toward the center of the bin. Consequently, the equilibrium horizontal distribution of the air pressure in Figure 11(b) and the convergence granular and air flows below the outlet are formed as shown in Figure 4 and Figure 10(a).

**Figure 12:**
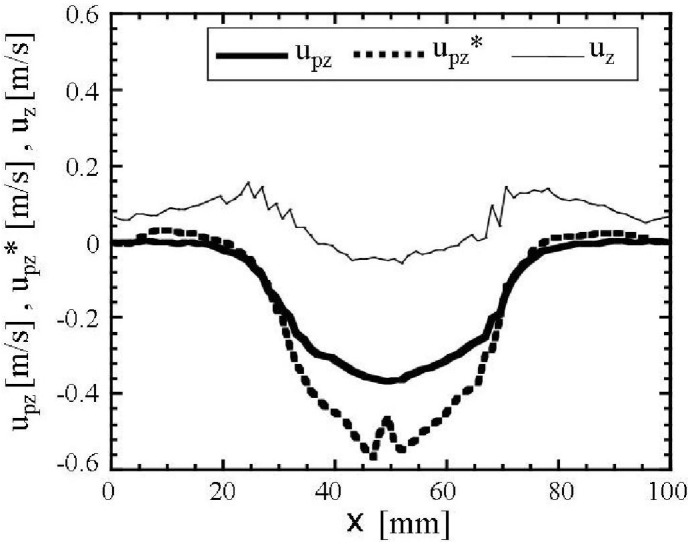
Calculated horizontal distributions of vertical granular velocities u_pz_ in the presence of air and u_pz_^*^ in a vacuum and vertical air velocity u_z_ at t = 0.2 s and 20 mm above the hopper outlet and y = 2.5 mm.

The descending granular velocity in the presence of air at 20 mm above the hopper outlet is smaller than that in a vacuum as shown in Figure 12. This figure also indicates that the direction of the air vertical velocity at the same height, 20 mm, from the hopper outlet is upward in the area between the plug flow zone and the side walls, and the direction becomes downward in the plug flow zone. The descending air velocity is much smaller than that of the granular flow. This means that the relative velocity between the granular particle and the air is large. These results suggest that the interaction between the granular motion by gravity and the quiescent air is large and causes the air motion and it reduces the descending granular velocity. The interaction between granular particles and air is mainly represented by Sp_i_ term in Equations 17 and 18. The interaction term Sp_z_ which acted on particles against gravity was about 50% of gravity term in Equation 17 in the central region (x = 20 mm~80 mm) at 20 mm height from the hopper outlet at t = 0.2 s after the opening of the slit. As the mean free path of the air at 10 Pa becomes about 10^4^ times longer than that at atmospheric pressure [50], the slip factor Cc in Equation 20 becomes about 12 times larger [42]. Thus, Sp_z_ is very small and the descending granular velocity becomes larger at the very low pressure, 10 Pa, than that in the atmospheric pressure. The calculated horizontal distributions of the packing fraction near the hopper outlet indicated that the bulk density in the presence of air is almost the same as that in a vacuum. Therefore, the reduced descending granular velocity by the air decreases the granular flow rate.

## 6. Conclusions

We have obtained the numerically empirical constitutive equations for the strain rate-independent stress as the functions of the Almansi strain including the large deformation by the same method as Yuu *et al.* [1,19]. The relations cover the elastic and plastic regions including the flow state. The non-linearity of the relations is considered by the functions of scalar of the Almansi strain tensor. The relations take account of the bulk density effect by the simple linear equation of state and represent the friction mechanism of granular material. We have presented simulation results of the evolution of granular flows of 200 μm particles and piles in a flat-bottomed hopper and bin in the presence of air and in a vacuum using our three-dimensional constitutive relations by SPH and two-way coupling methods. The granular flow patterns, the shapes of piles and the granular flow rates in the evolution in the presence of air were compared with the experimental data measured under the same conditions. The granular flow patterns and the shapes of piles in a vacuum were also compared with the experimental data. The good agreement between these results shows that the present constitutive relations and the simulation method would be applicable for granular quasistatics and dense granular dynamics in a hopper with complex flow geometry, including free surface. We have presented the effect of air on the granular flow of 200 μm particles in a hopper and a bin and shown that the air largely affects the granular flow of 200 μm particles in a flat-bottomed hopper and bin. These results have indicated the mechanism by which the air decreases the granular flow rate and the formation mechanism of the convergence granular flow below the hopper outlet by the interaction between the granular particles and air flows. The simulation results have indicated the formation mechanism of the granular flow rate peak occurred shortly after opening the slit.

## Figures and Tables

**Figure A1:**
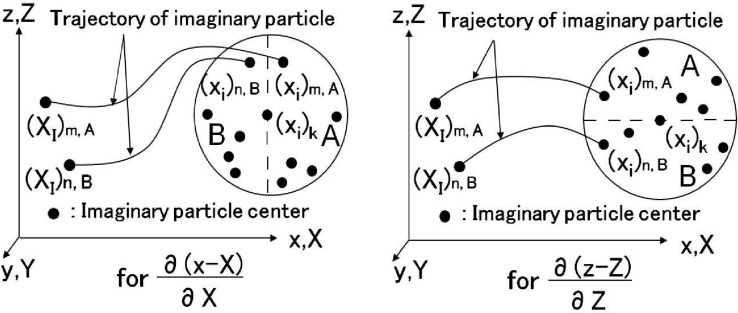
Example of spheres of 1.2h_k_ in radius, half spheres *(A* and *B*) and trajectory of imaginary particle for the calculation of the deformation gradient tensor **F**, the left Cauchy-Green tensor **B** and the Almansi strain tensor **Ea**.

**Figure A2:**
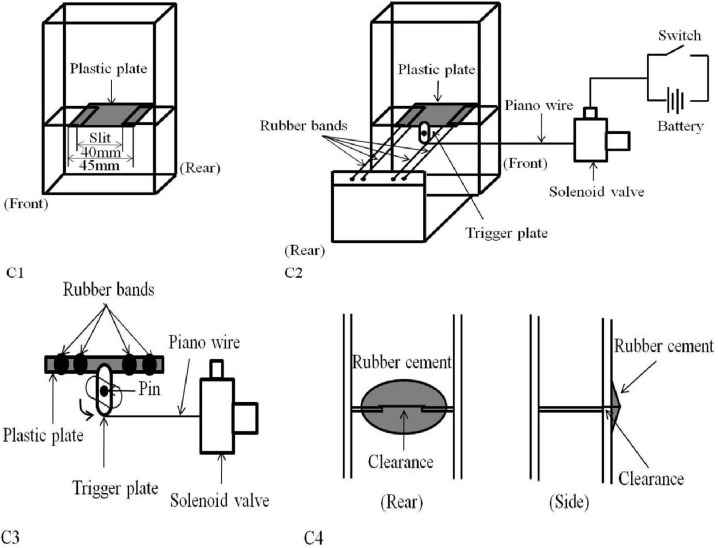
Apparatus for instant opening of slit.

## Supplementary Files

Supplementary File 1

## Appendix A: Calculation of Ea_ij_ in SPH

In the SPH calculation we labeled each imaginary particle with its initial coordinate X_I_ and advected the imaginary particles with the flow. We obtained x_i_ = x_i_ (X_I_, t) of these imaginary particles. We calculated the components of the deformation gradient tensor, F_iI_ = ∂ x_i_/ ∂ X_I_, and the left Cauchy-Green tensor, B_ij_ = ( ∂ x_i_/ ∂ X_I_)( ∂ x_j_/ ∂ X_I_), using the obtained x_i_ = *x*_i_(X_I_, t) and obtained the Almansi strain tensor Ea_ij_. The sphere of 1.2h_k_ in radius of which center is (x_i_)_k_ of the imaginary particle k was divided into two half spheres *A* and *B* in the direction x_i_ as shown in Figure A. Values (x_i_ − X_I_) and X_I_ of the imaginary particles existing in each half sphere were averaged. Usually 4~7 imaginary particles existed in each half sphere. We assumed that ∂ (x_i_ − X_I_) / ∂ X_I_ = [(x_i_ − X_I_) averaged in the half sphere *A* − (x_i_ − X_I_) averaged in the half sphere *B*]/[averaged X_I_ of imaginary particles in the half sphere *A* − averaged X_I_ of imaginary particles in half sphere *B*]. Other values, for example ∂ (x_j_ − X_J_)/ ∂ X_J_ and ∂ (x_i_ − X_I_) ∂ X_J_, were calculated by the same method. Using them, F_iI_ and B_ij_ were obtained and Ea_ij_ was obtained.

## Appendix B: Non-linear Functions of A1, A2, A3, and A4 in Constitutive Equations (1)–(6)

The fitting functions of A1, A2, A3 and A4 are as follows:

**Table:**

A1 = 9.4 × 10^13^a^2^ + 2.0 × 10^9^a + 9.0 × 10^5^, 0 ≤ a < 0.000045
A1 = −8.0 × 10^14^a^2^ + 8.0 × 10^10^a − 8.0 × 10^5^, 0.000,045 ≤ a < 0.00005
A1 = −1.2 × 10^17^a^3^ + 6.7 × 10^13^a^2^ − 1.4 × 10^10^a + 1.7 × 10^6^, 0.000,05 ≤ a < 0.0002
A1 = 1.2 × 10^12^a^2^ − 2.1 × 10^9^a + 1.1 × 10^6^, 0.0002 ≤ a < 0.0004
A1 = −3.6 × 10^14^a^3^ + 1.2 × 10^12^a^2^ − 1.5 × 10^9^a + 9.0 × 10^5^, 0.0004 ≤ a < 0.001
A1 = −3.8 × 10^12^a^3^ + 4.1 × 10^10^a^2^ − 2.0 × 10^8^a + 4.4 × 10^5^, 0.001 ≤ a < 0.0035
A1 = −1.4 × 10^12^a^3^ + 2.7 × 10^10^a^2^ − 1.6 × 10^8^a + 4.1 × 10^5^, 0.0035 ≤ a < 0.007
A1 = −6.2 × 10^8^a^3^ + 5.5 × 10^7^a^2^ − 2.1 × 10^6^a + 4.1 × 10^4^, 0.007 ≤ a ≤ 0.035
A2 = 9.4 × 10^13^a^2^ + 2.0 × 10^9^a + 3.0 × 10^5^, 0 ≤ a < 0.000045
A2 = −8.0 × 10^14^a^2^ + 8.0 × 10^10^a − 1.4 × 10^6^, 0.000,045 ≤ a < 0.000055
A2 = 7.8 × 10^16^a^3^ − 6.2 × 10^12^a^2^ − 4.8 × 10^9^a + 8.5 × 10^5^, 0.000,055 ≤ a < 0.00016
A2 = −7.0 × 10^15^a^3^ + 7.4 × 10^12^a^2^ − 2.6 × 10^9^a + 5.0 × 10^5^, 0.000,16 ≤ a < 0.00045
A2 = −2.3 × 10^14^a^3^ + 6.2 × 10^11^a^2^ − 5.7 × 10^8^a + 3.3 × 10^5^, 0.000,45 ≤ a < 0.001
A2 = 1.7 × 10^13^a^3^ − 9.5 × 10^10^a^2^ + 1.2 × 10^8^a + 1.0 × 10^5^, 0.001 ≤ a < 0.0025
A2 = −2.7 × 10^11^a^3^ + 6.9 × 10^9^a^2^ − 5.8 × 10^7^a + 2.0 × 10^5^, 0.0025 ≤ a < 0.007
A2 = 3.1 × 10^8^a^2^ − 8.5 × 10^6^a + 7.6 × 10^4^, 0.007 ≤ a < 0.012
A2 = 1.5 × 10^6^a^2^ − 5.7 × 10^5^a + 2.6 × 10^4^, 0.012 ≤ a ≤ 0.035
A3 = 7.3 × 10^12^a^2^ + 7.3 × 10^7^a + 3.0 × 10^5^, 0 ≤ a < 0.000165
A3 = −1.8 × 10^14^a^2^ + 6.2 × 10^10^a − 4.7 × 10^6^, 0.000,165 ≤ a < 0.000175
A3 = 1.5 × 10^16^a^3^ − 1.1 × 10^13^a^2^ + 5.7 × 10^8^a + 6.5 × 10^5^, 0.000,175 ≤ a < 0.0004
A3 = −1.2 × 10^14^a^3^ + 5.1 × 10^11^a^2^ − 7.4 × 10^8^a + 3.9 × 10^5^, 0.0004 ≤ a < 0.0016
A3 = −9.0 × 10^11^a^3^ + 1.1 × 10^10^a^2^ − 4.8 × 10^7^a + 7.4 × 10^4^, 0.0016 ≤ a < 0.004
A3 = −2.3 × 10^10^a^3^ + 1.5 × 10^9^a^2^ − 1.4 × 10^7^a + 3.2 × 10^4^, 0.004 ≤ a < 0.007
A3 = 6.7 × 10^9^a^3^ − 2.8 × 10^8^a^2^ + 3.9 × 10^6^a − 1.9 × 10^4^, 0.007 ≤ a < 0.015
A3 = −2.2 × 10^5^a^2^ + 4.4 × 10^4^a − 1.9 × 10^3^, 0.015 ≤ a ≤ 0.035
A4 = 4.6 × 10^10^a^2^ + 1.4 × 10^6^a + 1.5 × 10^3^, 0 ≤ a < 0.00011
A4 = −3.0 × 10^11^a^2^ + 7.2 × 10^7^a − 2.1 × 10^3^, 0.00011 ≤ a < 0.000,13
A4 = 3.1 × 10^12^a^3^ − 5.7 × 10^8^a^2^ − 3.7 × 10^6^a + 2.7 × 10^3^, 0.000,13 ≤ a < 0.00055
A4 = −3.9 × 10^11^a^3^ + 1.9 × 10^9^a^2^ − 3.2 × 10^6^a + 2.3 × 10^3^, 0.000,55 ≤ a < 0.0019
A4 = −2.7 × 10^9^a^3^ + 4.6 × 10^7^a^2^ − 2.8 × 10^5^a + 7.3 × 10^2^, 0.0019 ≤ a <0.006
A4 = −7.4 × 10^7^a^3^ + 3.1 × 10^6^a^2^ − 4.6 × 10^4^a + 3.0 × 10^2^, 0.006 ≤ a < 0.015
A4 = 6.9 × 10^4^a^2^ − 5.0 × 10^3^a + 1.1 × 10^2^, 0.015 ≤ a ≤ 0.035
where a = (Ea_ij_ Ea_ij_)^0.5^
